# Effects of some gelling agents and their concentrations on conversion of oil palm polyembryoids into plantlets

**DOI:** 10.1186/s43141-019-0018-z

**Published:** 2020-02-03

**Authors:** Sharrmila Rengeswari Palanyandy, Saikat Gantait, Uma Rani Sinniah

**Affiliations:** 1 Department of Crop Science, Faculty of Agriculture, Universiti Putra Malaysia, 43400 Serdang, Selangor Malaysia; 2 Crop Research Unit (Genetics and Plant Breeding), Bidhan Chandra Krishi Viswavidyalaya, Mohanpur, Nadia, West Bengal 741252 India

**Keywords:** Agar Type 900, Gelrite, Oil palm, Polyembryoids, Secondary somatic embryos

## Abstract

Oil palm, a tropical plant with an economic life of 20–25 years, is on high demand since its oil (palm oil) is now considered to be the world’s most consumed oil. Despite the high potential for the use of clonal materials, the tissue culture technique for oil palm is difficult and laborious. One of the key steps of the process is the conversion of polyembroids into plantlets. Gelling agent has been implicated to play a role in ensuring the conversion of oil palm polyembryoids into complete plantlets. In the present study, for the first time, we report the effects of two types of common gelling agents, Agar Type 900 and Gelrite®, for enhanced conversion of oil palm polyembryoids into plantlets. Polyembryoids, developed from embryonic calli, were cultured and incubated on Murashige and Skoog semisolid media supplemented with Agar (Type 900) at 8–12 g/l or gellan gum (Gelrite®) 1.5–3.5 g/l. The effects of gelling agents on polyembryoid conversion was assessed based on the percentages of viability, survival, and polyembryoids that swelled, enlarged, and turned green, as well as on the basis of morphological characteristics, viz, number of shoots, leaves, roots, secondary somatic embryos, and callus formation. Based on the results of this study, in comparison to Agar Type 900, the Gelrite® with 3.5 g/l concentration was chosen as an effective gelling agent for conversion of polyembryoids into plantlets, since it resulted in 100% survival with 53.3% completely developed plantlets (multiple shoots with roots). The successful conversion of polyembryoids into plantlets achieved in this study, using the optimized gelling agent could be useful for pre-storage or post-storage conversion in many other plant species as well.

## Introduction

Oil palm (*Elaies guineensis* Jacq.) is a tropical plant, popularly known as “African oil palm” due to its origin from the Gulf of Guinea in West Africa. It is a perennial crop with a productive life of longer than 50 years. However, economically, the life span of oil palm is about 20–25 years. Palm oil is now the world’s most consumed oil and is a major source for oleochemicals and biofuels. Due to the mounting demand for palm oil, traditionally, for those involved in the oil palm industry, increase in yield in terms of fresh fruit bunch and oil content is the main agenda [1]. Considerable yield increases would be possible only if genetically uniform plants from superior genotypes are established through the production of clonal palms. Despite the high potential for the use of clonal materials, the tissue culture technique for oil palm is difficult and laborious. One of the key factors in oil palm tissue culture that hinders the development of an economically viable propagation system is the use of suitable gelling agent that would enhance the conversion efficiency of oil palm polyembryoids into complete plantlets. High conversion of polyembryoids into plantlets is also a necessary step in establishing a protocol for oil palm tissue culture. The type of gelling agent used has a great influence on the growth of the tissue in culture [2]. Among the gelling agents, the concentration used also can generate variable results [3]. These effects may be the result of differences in the strength of the gel produced, mineral composition and/or availability, and the presence of inhibitory compounds [4].

The most common gelling agents that are generally used commercially are Agar Type 900 and gellan gum (Gelrite®). For the preparation of semisolid plant tissue culture media, agar is the most frequently used gelling agent. There are a number of benefits of using agar instead of employing other gelling agents. First, when agar is blended with water, it forms a gel that liquefies at ~ 60–100 °C and solidifies at ~ 45 °C; thus, agar gels are steady at all possible incubation temperatures. Furthermore, agar gels do not react with components of media and are not broken down by plant enzymes. The firmness of an agar gel is regulated by the concentration and brand of agar used in the culture medium and the pH of the medium [5]. The agar concentrations generally used in culture media vary within 0.5 and 1% (w/v); these concentrations result in semisolid gel at a typical pH of the media. Meanwhile, Gelrite®, an alternative to agar [6] is a synthetic product and generally supplemented at 1.25–2.5 g/l, ensuing in a transparent and consistent gel that furthermore assists in identifying any biotic contamination. Whether explants regenerate best on agar or on other supplementary agents, depends on species to species.

Till date, to the best of our knowledge, determination of suitable gelling agents in media for oil palm tissue culture has not been reported. Hence, the effects of two types of common gelling agents, viz, Agar Type 900 (Merck, Germany) and Gelrite® (Merck, Germany), for enhanced conversion of oil palm polyembryoids into plantlets was studied in the present experiment.

## Materials and methods

### Materials

The initial experimental material was in the form of oil palm (Clone 5074) cell suspension culture of a selection of Tenera (Dura × Pisifera) hybrid. Cell aggregates > 500 μm in size were sieved and placed in Murashige and Skoog [7] (MS) semisolid media (mentioned below) and incubated until induction of polyembryoids [8].

### Preparation of culture medium with different gelling agents

MS medium was used in all the media and solutions used for developing culture medium for oil palm. After MS basal medium was supplemented with 30 g/l sucrose, 100 mg/l myoinositol was dissolved and sterile distilled water was added to make up for the final required volume. The pH of the medium was adjusted to 5.75 with 0.1 M NaOH or HCl followed by addition of gelling agents (of different types and concentrations), prior to sterilization at 121 °C for 20 min. Six types of culture media (supplemented with Agar Type 900 at 8, 10, and 12 g/l or Gelrite® 1.5, 2.5, and 3.5 g/l) were prepared consistent with the essential treatments.

### Conversion of polyembryoids

The polyembryoids were incubated at 25 °C and 60 μmol/m^2^/s^2^ irradiance under standard cool fluorescent tubes (Philips Lifemax, PHILIPS, Indonesia) with 16 h photoperiod. Continuous subcultures were done at 30 days intervals in MS semisolid medium, and conversion of polyembryoids was witnessed periodically.

### Observation and data collection

The effects of gelling agents on the conversion of polyembryoids, based on percentage of viability and survival, was scored. The percentage of polyembryoids that swelled and enlarged in size was recorded as 'viable' after 10 days of culture. Meanwhile, the percentage of polyembryoids that turned green was recorded as 'survived' after 30 days of culture. Furthermore, morphological characteristics such as number of leaves, roots, secondary somatic embryos (SSE), and callus formation were recorded at 60 days of culture. At 90 days of culture, the percentages of multiple shoots with root formation, shoot formation only, root formation only, callus formation, SSE formation, and dead polyembryoids were recorded.

### Experimental design and statistical analysis

The experiment was laid out in completely randomized design comprising three replications per treatment, with each involving 20 polyembryoids. Collected data from the experiments were subjected to one-way analysis of variance (ANOVA) using Statistical Analysis Software version 9.2. All percent data were transformed to square root values before being subjected to ANOVA. Means were compared with Duncan’s multiple range tests [9] at *P* ≤ 0.05 level.

## Results and discussion

In the current study, the effects of two gelling agents, namely, Agar Type 900 and Gelrite®, with different concentrations was evaluated for their ability to influence the conversion of polyembryoids into plantlets based on survival percentage and morphological characterization. Conversion of polyembryoids was observed after the first month of incubation, but no significant treatment differences were discerned at that stage, based on viability scoring at 10 days of culture and survival scoring at 30 days of culture. The viability was scored as percentage of the polyembryoids that swelled and enlarged in size; on the other hand, survival was scored as percentage of polyembryoids that turned green. It could be clearly seen that polyembryoids that developed on 3.5 g/l Gelrite® turned green and exhibited the highest conversion (100%) into plantlets (Table 1). However, the effect of Gelrite® concentration on development was discernible even at the first scoring period, 2 months after the initiation of the experiment. Higher number of leaves, roots, and calli were obtained using 3.5 g/l Gelrite® concentration (Table 2). This result is in agreement with an earlier study on *Sequoia sempervirens* wherein it was found that Gelrite® resulted in a higher shoot multiplication [11].

**Table 1:** Effects of different type of gelling agents on the viability and survival of oil palm polyembryoids

Gelling agent	Concentration (g/l)	Viability (%)	Survival (%)
Gelrite®	1.5	100.0a	80.0d
	2.5	100.0a	90.0b
	3.5	100.0a	100.0a
Agar Type 900	8	86.7b	86.7c
	10	86.7b	80.0d
	12	86.7b	86.7c

Values in the same column having the different letters are significantly different at *P* ≤ 0.05 based on Duncan Multiple Range Test [9]. Data expressed as percentage were transformed using arc sine prior to ANOVA and converted back to the original scale for demonstration in the table [10]

**Table 2:** Effects of different type of gelling agents on the production of number of leaves, roots, SSE, and callus in oil palm polyembryoids after 60 days of culture

Gelling agent	Concentration (g/l)	No. of leaves	No. of roots	No. of SSE	No. of callus
Gelrite®	1.5	10.6c	2.7 cd	4.3a	0.0d
	2.5	13.0b	8.0a	0.0c	0.7c
	3.5	13.7a	5.3b	0.0c	1.7b
Agar Type 900	8	8.3d	2.3d	1.0b	1.3bc
	10	12.3bc	5.3b	2.6ab	2.0a
	12	10.0 cd	3.0c	0.0c	0.3 cd

Values in the same column having the different letters are significantly different at *P* ≤ 0.05 based on Duncan Multiple Range Test [9]

**Fig. 1:**
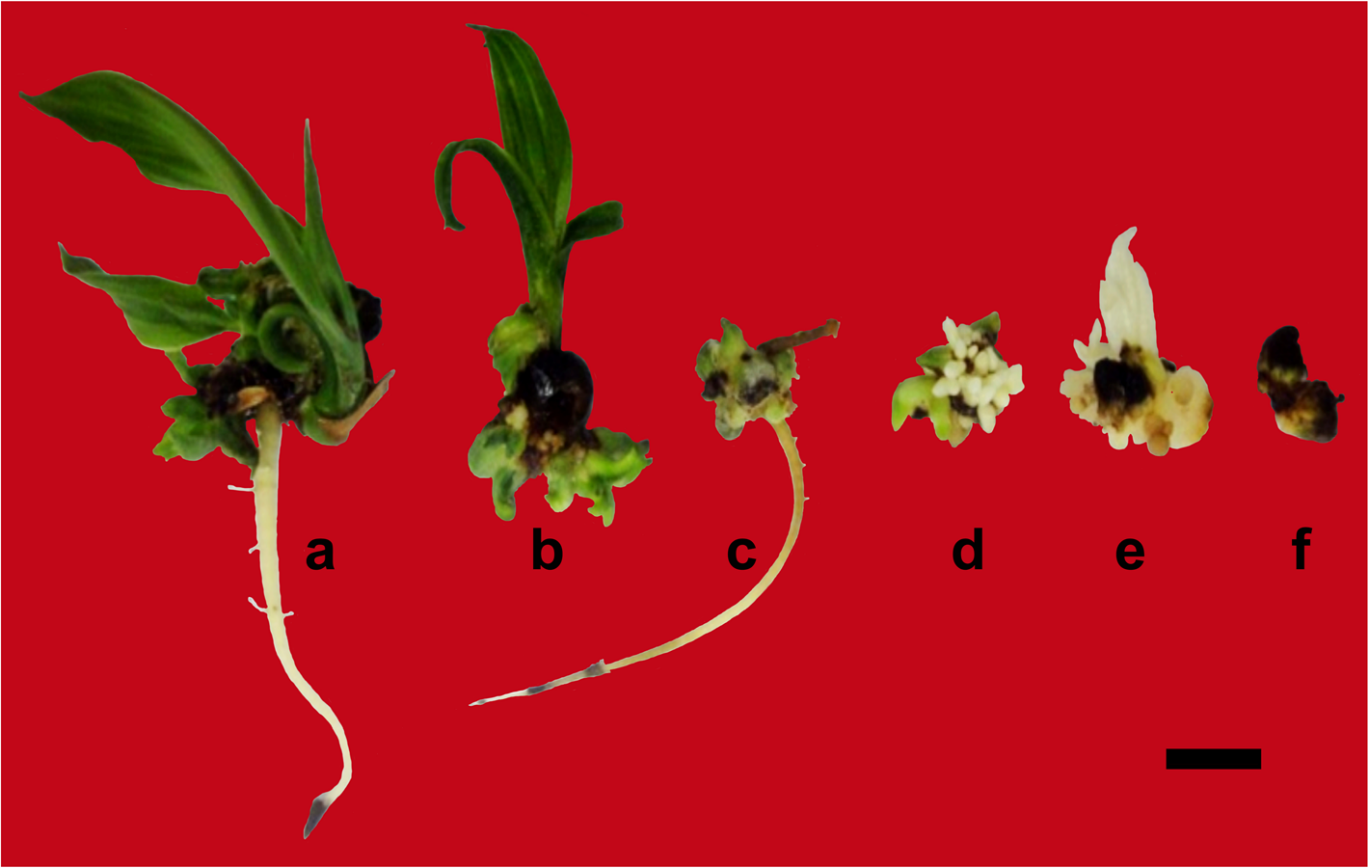
Influence of different gelling agents (supplemented in MS medium) on conversion and morphological characteristics of oil palm polyembryoids. **a** Induced multiple shoots with root in 3.5 g/l Gelrite®. **b** Induced shoots (only) in 8 g/l Agar Type 900. **c** Induced root (only) in 12 g/l Agar Type 900. **d** Induced secondary somatic embryos in 1.5 g/l Gelrite®. **e** Induced callus in 2.5 g/l Gelrite®. **f** Dead (degenerated) polyembryoid in 10 g/l Agar Type 900 (bar = 5 mm)

Somewhat similar developmental profiles were seen at the 3-month growth stage (90 days) when the highest percentage of multiple shoots with root (53.3%) was found in the culture medium having 3.5 g/l Gelrite® (Table 3). The different morphological characteristics observed after 90 days of culture is shown in Fig. 1. It was observed that better plantlet formation was obtained using 3.5 g/l Gelrite®, which evidently led to less formation of plantlets with shoot or root only (26.7% and 6.7%, respectively). It was further noticed that a similar percentage of multiple shoots with root (53.3%) was produced in 1.5 g/l Gelrite® concentration, but 20% of dead polyembryoids were recorded using this treatment; vice versa, no dead polyembryoids were observed in 3.5 g/l Gelrite®. It was evident from an earlier report that water availability and nutrients uptake were significantly affected by nature and strength of the gelling agent and also by the interaction between the explants and the matrix [12]. Hence, in the present study, 3.5 g/l Gelrite® (due to its inert nature for water uptake) seems to have better nutrient uptake and water availability compared to other treatments.

**Table 3:** Effect of different type of gelling agent on oil palm polyembryoids morphology after 90 days of culture

Gelling agent	Concentration (g/l)	Multiple shoot with root (%)	Shoot only (%)	Root only (%)	Callus (%)	SSE (%)	Dead (%)
Gelrite®	1.5	53.3a	6.7d	0.0c	6.7b	13.3a	20.0a
	2.5	46.7b	20.0c	13.3b	13.3a	6.7b	0.0c
	3.5	53.3a	26.7b	6.7b	0.0c	13.3a	0.0c
Agar Type 900	8	33.3d	33.3a	13.3b	0.0c	6.7b	13.3b
	10	40.0c	33.3a	0.0c	0.0c	6.7b	20.0a
	12	26.7e	33.3a	20.0a	6.7b	0.0c	13.3b

Values in the same column having the different letters are significantly different at *P* ≤ 0.05 based on Duncan Multiple Range Test [9]. Data expressed as percentage were transformed using arc sine prior to ANOVA and converted back to the original scale for demonstration in the table [10]

Overall, Gelrite® was proven to be better than Agar Type 900, giving the highest conversion rate into plantlets formation over several months of culture. Comparatively, Agar Type 900 resulted in poor levels of conversion into plantlets, and Gelrite® was confirmed to be the optimum gelling agent for polyembryoids growth and development that was best at 3.5 g/l concentration, since the highest percentage of multiple shoots with roots was obtained using this treatment. It might be due to the fact that Gelrite® 3.5 g/l have better interaction for nutrient and water uptake for the conversion of polyembryoids into plantlets by providing superior substrate in optimized concentration.

It is known that gelling agents supplemented in culture medium play an important role in the growth and development of plant cultured in vitro. Usually, the concentration of gelling agents exhibits a direct association with water stress. It was reported earlier that a very high concentration of gelling agent resulted in high water stress which led to the difficulty of uptaking water and nutrient from culture medium [4]. Many authors reported the influence of gelling agents on the development of adventitious shoots, calli [13, 14], somatic embryos [2, 3, 15], and protoplasts [16] under in vitro culture. These studies showed that the both type and quality of gelling agents also create problems related with hyperhydricity and necrosis of the tissue. The difference in the quality of gelling agents from brand-to-brand and also batch-to-batch may occur. However, it is still unclear whether gelling agents which even though successfully perform well with several plant species could be effective for a particular plant of interest.

## Conclusion

In this study, it was identified that 3.5 g/l Gelrite® was the most effective gelling agent for enhanced conversion of polyembryoids into plantlets, since it brought about 100% survival with 53.3% completely developed plantlets (multiple shoots with roots). The successful conversion of polyembryoids into plantlets, achieved in this study, using selected suitable gelling agent and its concentration, could be useful for pre-storage or post-storage conversion experiments for many other plant species in near future.

## Data Availability

The datasets used and/or analyzed during the current study are available from the corresponding author on reasonable request.

## Abbreviations

ANOVA: Analysis of variance
MS: Murashige and Skoog
SSE: Secondary somatic embryos
